# Impaired Expression of Neuregulin 1 and Nicotinic Acetylcholine Receptor β4 Subunit in Diverticular Disease

**DOI:** 10.3389/fncel.2019.00563

**Published:** 2019-12-19

**Authors:** Martina Barrenschee, François Cossais, Martina Böttner, Jan-Hendrik Egberts, Thomas Becker, Thilo Wedel

**Affiliations:** ^1^Neurogastroenterology, Institute of Anatomy, Christian-Albrechts University of Kiel, Kiel, Germany; ^2^Department of General, Visceral-, Thoracic-, Transplantation-, and Pediatric Surgery, University Hospital Schleswig-Holstein, Kiel, Germany

**Keywords:** NRG1, HRG 1 beta, nAchR, enteric nervous system, diverticular disease, ErbB2, ErbB3

## Abstract

Neuregulin 1 (NRG1) regulates the expression of the nicotinic acetylcholine receptor (nAChR) and is suggested to promote the survival and maintenance of the enteric nervous system (ENS), since deficiency of its corresponding receptor complex ErbB2/ErbB3 leads to postnatal colonic aganglionosis. As diverticular disease (DD) is associated with intestinal hypoganglionosis, the NRG1-ErbB2/ErbB3 system and the nAChR were studied in patients with DD and controls. Samples of tunica muscularis of the sigmoid colon from patients with DD (*n* = 8) and controls (*n* = 11) were assessed for mRNA expression of NRG1, ErbB2, and ErbB3 and the nAChR subunits α3, α5, α7, β2, and β4. Site-specific gene expression levels of the NRG1-ErbB2/3 system were determined in myenteric ganglia harvested by laser microdissection (LMD). Localization studies were performed by immunohistochemistry for the NRG1-ErbB2/3 system and nAChR subunit β4. Rat enteric nerve cell cultures were stimulated with NRG1 or glial-cell line derived neurotrophic factor (GDNF) for 6 days and mRNA expression of the aforementioned nAchR was measured. NRG1, ErbB3, and nAChR subunit β4 expression was significantly down-regulated in both the tunica muscularis and myenteric ganglia of patients with DD compared to controls, whereas mRNA expression of ErbB3 and nAChR subunits β2, α3, α5, and α7 remained unaltered. NRG1, ErbB3, and nAChR subunit β4 immunoreactive signals were reduced in neuronal somata and the neuropil of myenteric ganglia from patients with DD compared to control. nAChR subunit β4 exhibited also weaker immunoreactive signals in the tunica muscularis of patients with DD. NRG1 treatment but not GDNF treatment of enteric nerve cell cultures significantly enhanced mRNA expression of nAchR β4. The down-regulation of NRG1 and ErbB3 in myenteric ganglia of patients with DD supports the hypothesis that intestinal hypoganglionosis observed in DD may be attributed to a lack of neurotrophic factors. Regulation of nAChR subunit β4 by NRG1 and decreased nAChR β4 in patients with DD provide evidence that a lack of NRG1 may affect the composition of enteric neurotransmitter receptor subunits thus contributing to the intestinal motility disorders previously reported in DD.

## Introduction

Diverticular disease (DD) is one of the most common diseases in western countries with high prevalence especially in the elderly. It is characterized by multiple mucosal/submucosal herniations (pseudo-diverticula) through the colonic muscle coat which can lead to a broad spectrum of symptoms with potentially lethal complications (Jun and Stollman, 2002). Despite the considerable burden of DD on the healthcare system (Sandler et al., 2002), the pathogenesis of DD is still discussed controversially and appears to be multifactorial. Increasing age, low-fiber diet, and connective tissue alterations are considered traditionally as risk factors for the formation of colonic diverticula (Strate et al., 2009; Böttner and Wedel, 2012). Moreover, previous reports have given evidence for an underlying enteric neuropathy in DD characterized by a decrease of myenteric nerve cells (oligoneuronal hypoganglionosis) and reduced nerve fibers within smooth muscle layers (Golder et al., 2003; Iwase et al., 2005; Deduchovas et al., 2008; Wedel et al., 2010). It is postulated that the disturbed innervation gives rise to colonic motility disorders frequently reported in DD (Bassotti et al., 2001) thereby promoting the development of diverticula.

Although a loss of enteric neurons represents a common histopathologic phenotype within the spectrum of gastrointestinal neuromuscular pathology (Knowles et al., 2010), the pathomechanisms leading to the reduced ganglionic nerve cell content observed in DD remain unclear. Moreover, the underlying cellular and molecular mechanisms leading to DD are largely unknown (Simpson and Spiller, 2002). However, since DD is associated with a lack of glial-cell line derived neurotrophic factor (GDNF) and its corresponding receptors (Böttner et al., 2013), a general involvement of neurotrophic factors may play a role in the pathogenesis of DD, e.g., a deficient GDNF system could be a primary trigger for the reduced neuronal number shown in DD (Barrenschee et al., 2017).

Neuregulin 1 (NRG1) belongs to a family of growth factors that contain an epidermal growth factor domain and activates the ErbB2/ErbB3 tyrosine kinases in neural crest cells (Britsch et al., 1998; Garratt et al., 2000). NRG1 is crucial for various functions in the nervous system, e.g., nerve cell differentiation, neurite outgrowth, and synapse formation (Falls, 2003; Mei and Nave, 2014). Isoforms of the **neuregulin 1** gene can be classified into three groups (type I–III) (Falls, 2003), where type I isoforms [neu differentiation factor (NDF); heregulin (HRG); acetylcholine receptor inducing activity (ARIA)] have been implicated in neuromuscular junction formation by stimulating muscular nicotinic acetylcholine receptor (nAChR) expression during development (Martinou et al., 1991; Falls et al., 1993).

The importance of NRG1 and its receptor signaling system ErbB2/ErbB3 in the enteric nervous system (ENS) became evident, when NRG1 or its corresponding receptors were ablated in animals. Immunoglobulin-like-*NRG1* deficient mice were found to show approximately 50% reduction in the number of AChRs at neuromuscular synapses and reduced synaptic strength (Sandrock et al., 1997). Additionally, ErbB3^–/–^ mice exhibited a total loss of enteric glia and reduced ganglionic number in the duodenum (Erickson et al., 1997; Riethmacher et al., 1997), and enteric neurons and glial cells were dramatically reduced in conditional ErbB2/Nestin-Cre mutant mice displaying an Hirschsprung’s disease-like phenotype (Crone et al., 2003). Recently, we could demonstrate that NRG1 acts as a neurotrophic factor for the ENS, since it promotes the growth and differentiation of postnatal enteric neurons (Barrenschee et al., 2015).

Excitatory nicotinic cholinergic transmission is essential for the regulation of gastrointestinal motility and mediated by neuronal nAChRs in the ENS (Galligan and North, 2004). nAChRs are ligand-gated pentameric ion channels found both in the central and peripheral nervous systems composed of various combinations of alpha and non-alpha subunits (Sargent, 1993; Dani and Bertrand, 2007) resulting in specific nAChR subtypes. In the mammalian nervous system, eight ligand binding α (α2–α7, α9–α10) and three structural β (β2–β4) subunits have been identified. Neuronal nAChRs are widely expressed in the central and peripheral nervous systems in adults and during development (Albuquerque et al., 2009). Whereas in the central nervous system the majority of nAChRs are either heteromeric α4β2 containing nAChRs or homomeric α7 nAChRs, in the peripheral nervous system heteromeric α3β4-type nAChRs with or without α5 are the predominant receptors (Mao et al., 2006). Although the expression of nAChRs in the ENS is less well established, it is known that enteric neurons contain α3, α5, α7, β2, and β4 subunit proteins which form different heteromeric nAChR subtypes (Obaid et al., 2005; Grundy and Schemann, 2006; Mandl and Kiss, 2007). In the peripheral nervous system, α3β4-type nAChRs are considered to represent the predominant receptors (Galligan and North, 2004).

Since DD is associated with a partial loss of enteric neurons and a lack of the nerve growth factor GDNF with concomitant intestinal motility disturbances, we raised the question whether the NRG1 growth factor system and the enteric nAchR subunit composition are also altered in DD, thereby contributing to the intestinal hypoganglionosis and motility dysfunctions in DD.

## Materials and Methods

### Patients

#### Control Group

Segments of sigmoid colon were obtained from patients (*n* = 10, four females, six males, mean age: 69.0 years) who underwent anterior rectal resection or left hemicolectomy for non-obstructive colorectal carcinoma. Anorectal evacuation and colonic motility disorders were previously excluded. None of the patients showed colonic diverticula. Full-thickness specimens were harvested from the sigmoid colon at safe distance (>5 cm) from the tumor.

#### Patients With DD

Segments of sigmoid colon were obtained from patients (*n* = 8, two females, six males, mean age: 62.0 years) who underwent sigmoid resection or left hemicolectomy for symptomatic DD (e.g., left-sided abdominal pain, meteorism, altered bowel habits). Patients were operated by elective surgery during symptom-free interval after two or more episodes of diverticulitis. Time between last episode and surgery ranged from 4 weeks to several months. Two patients showed non-obstructing stenotic narrowing of the sigmoid colon and one patient a covered perforation with paracolic abscess. Patients with emergency surgery for peritonitis due to open perforation or with complications due to obstructed stenosis and fistula formation have been excluded from the study. Full-thickness specimens were harvested from sites at a distance >1 cm from diverticula. Diverticula-containing areas displaying an altered anatomy of the colonic wall due to transmural mucosal herniation or fibrotic/inflammatory processes were excluded from tissue sampling. The study of human tissue received approval from the Local Ethics Committee of the Faculty of Medicine, Christian-Albrechts University of Kiel, Germany (B299/07).

### Tissue Preparation

#### Tissue Processing for mRNA Expression Profiles of the Tunica Muscularis and LMD Samples

The tunica muscularis was isolated from full-thickness biopsies of the colonic wall, immediately frozen in isopentane and stored at −70°C until use. Prior to RNA isolation 20 orthogonal cryosections (10 μm) were cut on a cryostat and collected in RNA lysis buffer (Macherey and Nagel, Düren, Germany). For isolation of myenteric ganglia orthogonal cryosections (10 μm) from full-thickness biopsies were placed on membrane-coated (polyethylene naphtalate, 1.0 μm, Zeiss, Göttingen, Germany) slides. To visualize myenteric ganglia sections were ultra-rapidly (ca. 60 s) stained with toluidine blue and air-dried.

#### Tissue Processing for Immunohistochemical Studies

After surgical removal, all specimens were transferred into phosphate-buffered saline (PBS) (pH 7.2) at 37°C to allow adaption and further dissection. Full-thickness rectangular tissue blocks (30 mm × 10 mm) were pinned out flat on a cork plate by fine needles without artificial stretching nor shortening, thereby preserving the original size. The longer border of the tissue block was orientated perpendicular to the gut axis and corresponded to the cutting surface for histologic sections, so that myocytes of the circular muscle layer were cut along their longitudinal axis. After fixation (4% paraformaldehyde in PBS) for 24 h and dehydration tissue blocks were transferred into paraffin wax and cut in sections (6 μm) for immunohistochemistry.

### Laser Microdissection and Pressure Catapulting

Laser microdissection (LMD) was performed by a modified method described previously (Böttner et al., 2010). Briefly, myenteric ganglia and smooth muscle cells of the tunica muscularis were identified by inverse light microscopy (Zeiss Axio Observer Z1, Zeiss, Göttingen, Germany), excised by LMD, and collected by laser pressure catapulting (PALM MicroLaser System, Zeiss, Göttingen, Germany) in caps of 0.5 mL reaction tubes. Ganglionic and smooth muscle tissue areas of 2 mm^2^ per specimen were collected, immediately dissolved in 350 μL RNA lysis buffer (Macherey and Nagel, Düren, Germany), and stored at −70°C until further use.

### RNA Extraction and Reverse Transcription

Extraction of total RNA from human tissue was performed using a Nucleospin II kit (Macherey and Nagel, Düren, Germany); RNA from enteric nerve cell cultures was isolated using a Nucleospin XS kit (Macherey and Nagel, Düren, Germany) according to the manufacturer’s guidelines. Prior to reverse transcription, contaminating genomic DNA was digested in a volume of 15 μL using 1.5 U of DNAse I (Sigma, Munich, Germany). Reverse transcription was carried out in a total volume of 30 μL containing 200 ng RNA, 375 ng random hexamer primer (GE Healthcare, Freiburg, Germany), 0.5 mM dNTPs (Promega, Mannheim, Germany), 0.01 M DTT, 1× reaction buffer, and 150 U Superscript II Reverse Transcriptase (Invitrogen, Karlsruhe, Germany). The annealing, elongation, and denaturation steps were carried out at 25°C for 10 min, at 42°C for 50 min, and at 70°C for 15 min, respectively.

### Quantitative PCR

Quantitative PCR (qPCR) reactions were run on an ABI Prism 7700 Sequence Detection System (TaqMan, Applied Biosystems, Foster City, CA, United States). Amplification reactions were carried out in a 20 μL volume containing 1× qPCR Master Mix Plus (Eurogentec, Cologne, Germany), 900 nM primers, 225 nM hybridization probe, and 2 μL cDNA. Samples were run in duplicate and amplified over 45 cycles. Each cycle consisted of a denaturation phase of 15 s at 95°C and a hybridization/elongation phase of 1 min at 60°C. mRNA expression profiles were measured for NRG1, ErbB2, ErbB3, nAchRb2, nAchRb4, nAchRa3, nAchRa5, nAchRa7, and the housekeeping gene HPRT in human samples, and for nAchRb2, nAchRb4, nAchRa3, nAchRa5, nAchRa7, and *hprt* in rat enteric cell cultures. Forward and reverse primers and probes are listed below.

#### Primers Amplifying Human Sequences

*NRG1 type I HRG*β*1* (NM_013956.3): forward primer: *5*′*-atggaggcggaggagctgta-3*′, reverse primer: *5*′*-ttgcagtaggccaccacaca-3*′, probe: *5*′*-tgaccataaccggcatctgcatcgc-3*′; *ErbB2* (NM_004448.2): forward primer: 5′-ggaagtacacgatgcggagact-3′, reverse primer: 5′-tctctttcaggatccgcatctg-3′, probe: 5′-tggagccgctgacacctagcgga-3′; *ErbB3* (NM_001982.3): forward primer: 5′*-tgccatcttcgtcatgttgaac*-3′, reverse primer: 5′-*tcaatataaacaccccctgacagaa*-3′, probe: 5′-agctccgcttgactcagctcaccga; *nAchRb2* (NM_000748), forward primer: 5′-*tgaggcgataatcttcccactc*-3′, reverse primer: 5′-*gcttatgg tgtcactggccc*-3′, probe: 5′-*tcagtgtgcatgagcgggagcaga*-3′, *nAchRb4* (NM_000750) forward primer: 5′-*caaccagatgcgctttgca-*3′, reverse primer: 5′*-tctggctgaaacaggaatgga*-3′, probe: 5′-*cctggaaca gctcccgctacgaggg*-3′, *nAchRa3* (NM_000743): forward primer 5′-*ccgacatcacatactcgctgtaca*-3′: reverse primer: 5′-*gacgagcacag tgaggaagga*-3′, probe: 5′-*ccggcgcctgcccttgttctaca*-3′, *nAchRa5* (NM_000745): forward primer: 5′-*aaccgtcttcgctatcaacattca*-3′, reverse primer: 5′-*gcagtttgggaagcgtgtga*-3′, probe: 5′-*catggcgccttt ggtccgcaag*-3′, *nAchRa7* (NM_000746): forward primer: 5′-*agaa tgggacctagtgggaatcc*-3′, reverse primer: 5′-*ggcgcatggtcactgtgaa*-3′, probe: 5′-*tgctgcaaagagccctaccccgatg*-3′, *HPRT* (NM_000194.2, house-keeping gene): forward primer: 5′-*tgaacgtcttgctcga gatgtg*-3′, reverse primer: 5′-*ccagcaggtcagcaaagaattt*-3′, probe: 5′-*tgggaggccatcacattgtagcc*-3′.

#### Primers Amplifying Rat Sequences

*NRG1 type I HRG*β*1* (AY973244.1) forward primer: 5′-*ctac cagaagagggtgctgacaa*-3′, reverse primer: 5′-*gccgctgcttcttggtttt*-3′, probe: 5′-*ctgctggtggtcggcatcttgtgtg*-3′; *ErbB2* (NM_017003.2): forward primer: 5′-*gctgctgcaggaaactgagttag*-3′, reverse primer: 5′-*ccttccttagctccgtctcttttag*-3′, probe: 5′-*ctgacgcccagcggagcaatgc*-3′; *ErbB3* (XM_006240755.1) forward primer: 5′-*cgaggagatgcgag ctttcc*-3′, reverse primer: 5′-*aaagcctgctgtgccagtaatc*-3′, probe: 5′-*ccccatgttcgttatgcccgcct*; *nAchRb2* (NM_019297.1), forward primer: 5′-*cattcgtcgcaaaccactcttc*-3′, reverse primer: 5′-*ccacagt ctgagggcaggtaga*-3′, probe5′-*ctgcgtactcatcacctcgctggcc-*3′, *nAchRb4* (NM_052806.2) forward primer: 5′-*atggtgcccagaatacacacaaa-*3′, reverse primer: 5′-*gaccgagatcaaagtgtcatcga*-3′, probe: 5′-*agttc gtcgcgatggttgttgaccg*-3′, *nAchRa3* (NM_052805.2): forward primer 5′-*attggaagtacgttgccatggt*-3′: reverse primer: 5′-*gccatcaag ggttgcagaaa*-3′, probe: 5′-*tggtgtgcattttaggaacggcggg*-3′, *nAchRa5* (NM_017078.2): forward primer: 5′-*gcacgaaaacagttgtcaggtaca*-3′, reverse primer: 5′-*gatcaaacgggaaaaaggtaacg*-3′, probe: 5′-*ctgtc acgtggacgcaaccagcaaa-*3′, *nAchRa7* (NM_012832.3): forward primer: 5′-*atgccacgttccacaccaat*-3′, reverse primer: 5′-*ccagcgaa cgtcaatgtagca*-3′, probe: 5′-*tctccctccaggcatattcaagagc*-3′, *hprt* (NM_012583.2, house-keeping gene): forward primer: 5′-*cgccag cttcctcctcaga*-3′, reverse primer: 5′-*ggtcataacctggttcatcact-*3′, probe: 5′-*ttttcccgcgagccgaccgg*-3′.

### Enteric Nerve Cell Cultures

Preparation of myenteric nerve cells was performed according to a method described previously (Schäfer et al., 1997). Briefly, after removing the small intestine from newborn Wistar rats (postnatal day 2–3), the tunica muscularis was stripped from the mucosa, followed by incubation for 2 h at 37°C in Ca^2+^- and Mg^2+^-free Hanks’ Balanced Salt Solution (HBSS, Gibco Life Technologies/Invitrogen, Karlsruhe, Germany) with antibiotics containing 1 mg/mL collagenase (Sigma, Munich, Germany). Afterward, fragments of myenteric plexus were collected under stereomicroscopic control and incubated for 15 min at 37°C in trypsin/EDTA (0.125 mg/mL Gibco, Life Technologies, Germany) to dissociate the plexus. The procedure was stopped by replacing trypsin/EDTA with fetal calf serum (FCS, Gibco, Life Technologies/Invitrogen, Karlsruhe, Germany). The cells were triturated, counted, and seeded at a density of 100,000 cells/mL on poly-D-Lysin-(Sigma)/Laminin-(Sigma, Munich, Germany) coated coverslips for immunocytochemistry studies or 12-well-plates for gene expression studies. Cells were incubated in defined medium consisting of Neurobasal A (Gibco, Life Technologies/Invitrogen, Karlsruhe, Germany) and B27 supplement (Gibco, Life Technologies/Invitrogen, Karlsruhe, Germany). Additionally, recombinant human NRG1-β1 (Thr176-Lys246, EGF Domain) (R&D Systems, Minnesota, MN, United States) or recombinant rat GDNF (Peprotech, Hamburg, Germany) was added to a final concentration of 0 (control), 2, or 10 ng/mL for NRG1-β1 or 50 ng/mL for GDNF. Cells were cultured for 1 week and medium was changed every second day.

### Immunohistochemistry

Immunoreactive signals were visualized using the avidin–biotin complex system (Vectastain Elite ABC Kit, Vector Laboratories, Burlingame, CA, United States). Briefly, paraffin-embedded tissue sections were incubated with 3% hydrogen peroxide to block endogenous peroxidase activity, rinsed in TBS-buffer (TRIS-buffered saline; 10 mM TRIS, 50 mM NaCl, pH 7.4), and pretreated with citrate buffer (pH 6.0, 95°C water bath, 20 min for ErbB2 and ErbB3, microwave, 2 × 750nm, 5 min for nAchRβ4). Thereafter, samples were incubated overnight with a polyclonal rabbit-anti-NRG1 β1 antibody (HRG β1, 1:500^1^, Aachen, Germany; immunogen sequence: KKPGKSELRINKAS), polyclonal rabbit-anti-ErbB2 antibody (1:1000^1^, Aachen, Germany), polyclonal rabbit-anti-ErbB3 antibody (1:2000^1^, Aachen, Germany), or polyclonal goat-anti-nAChRβ4 antibody (1:500 abcam, Cambridge, Germany; immunogen sequence: DYRLTWNSSRYEGVN), diluted in antibody diluent (Invitrogen, Karlsruhe, Germany), and incubated for 45 min with biotinylated goat anti-rabbit IgG (1:400, DAKO, Hamburg, Germany). After washing three times with TBS, sections were incubated for 45 min with an avidin–biotin complex (Vectastain ABC Standard, Vector Laboratories, Burlingame, CA, United States) conjugated with horseradish peroxidase. 3, 3′-diaminobenzidine (DAKO, Hamburg, Germany) was used as substrate chromogen. Sections were counterstained with Meyer’s hematoxylin. Omission of the primary or secondary antibody served as negative controls. Analysis was carried out with a light optical microscope (Nikon 6000, Nikon, Tokyo, Japan) coupled to a digital camera (Digital Sight, Nikon, Tokyo, Japan). Data acquisition was performed with NIS-Elements BR 3.2 software (Nikon, Tokyo, Japan).

### Quantitation of Immunofluorescence Signals

Image acquisition, processing, and analysis were performed as previously described (Barrenschee et al., 2017). Briefly, fluorescence immunohistochemical signals of myenteric ganglia (*n* = 8) were recorded in specimens of controls and patients with DD. Recordings were repeated for each myenteric ganglion in a corresponding blank specimen after omission of the primary antibody maintaining the same settings (e.g., magnification, exposure time for antibodies, DAPI staining). All recordings were performed using a light microscope (Axiovert 200 M, Zeiss, Germany) coupled to a digital camera [AxioCam MR3 (monochrome), Zeiss, Germany]. Axiovision (version 4.7, Zeiss, Germany) was used for displaying, managing, and storing the pictures. Image processing was carried out in ImageJ. Pictures were transformed to 8 bits, scaled and corrected for uneven illumination using the background correction plugin from Terry Wu available at the ImageJ website^2^ with iteration of 3 and radius of 6 ptx. Each myenteric ganglion was marked and the mean gray value (integrated density/μm^2^ ganglionic tissue) was determined. Eight ganglia per specimen were analyzed to calculate the mean gray value for each specimen. Background correction was calculated using the formula: Corrected mean gray value = mean gray value (target specimen) − mean gray value (blank specimen). Data were normalized to the experimental control and presented as fold increase of mean gray value.

### Statistical Analysis

Comparison of mRNA expression levels of the NRG1 system and nAchR subunits between the control group and patients with DD were carried out by using unpaired student’s *t*-test with Welch’s correction for unequal variances. Fluorescence quantification data were analyzed by unpaired student’s *t*-test; the effects of NRG1 and GDNF treatment on rat enteric nerve cell cultures regarding gene expression studies were analyzed by one-way-ANOVA followed by Bonferroni’s multiple comparison test (Prism^TM^, GraphPad, San Diego, CA, United States). Significant outlier was calculated with Grubb’s test (Prism^TM^, GraphPad, San Diego, CA, United States) and removed from the analysis. Differences were considered significant if *p* < 0.05 and displayed as ^∗^, whereas ^∗∗^ indicates *p* < 0.01 vs. control and ^∗∗∗^ = *p* < 0.001 vs. control. Results are expressed as mean ± SEM.

## Results

### Gene Expression Profiles of the NRG1 System and nAchR Subunits in Patients With DD and Controls

**FIGURE 1:**
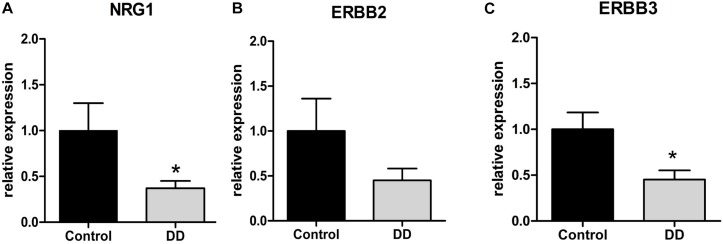
mRNA expression of the NRG1 system in the tunica muscularis of controls and patients with DD. mRNA expression of NRG1 and ErbB3 is significantly down-regulated in patients with DD compared to controls (**A,C**), whereas the reduction of ErbB2 mRNA levels is not statistically significant **(B)**. mRNA levels are determined by qPCR; expression of target genes is normalized to mRNA expression of the house-keeping gene HPRT. Data are shown as mean ± SEM, **n** = 6–9 per experimental group, ^∗^**p** < 0.05 vs. control.

To determine the gene regulation of the NRG1 system in patients with DD compared to controls, mRNA expression levels of NRG1 and its corresponding receptors ErbB2 und ErbB3 were monitored by qPCR. Analysis of the tunica muscularis revealed that NRG1 mRNA expression was significantly down-regulated in patients with DD, as mRNA levels dropped to 37 ± 8% of control values (Figure 1A). The NRG1 receptor ErbB3 also exhibited significant down-regulation with mRNA expression levels dropped to 45 ± 10% of control values (Figure 1C). mRNA expression of ErbB2 also decreased in patients with DD, however, not at significant level (Figure 1B).

**FIGURE 2:**
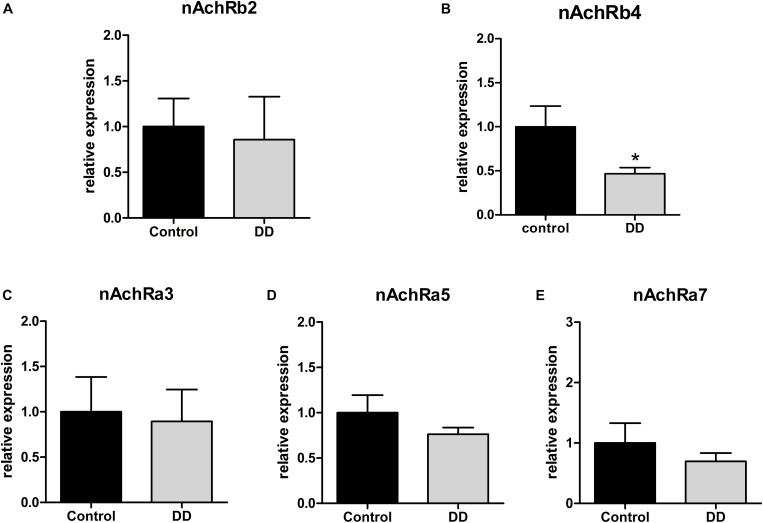
mRNA expression of nAchR subunits in the tunica muscularis of controls and patients with DD. mRNA expression of nAchRb4 is significantly down-regulated in patients with DD compared to controls **(B)**, whereas mRNA expression levels of nAchR subunits b2, a3, a5, a7 levels showed no significant difference compared to controls **(A,C,D,E)**. mRNA levels are determined by qPCR; expression of target genes is normalized to mRNA expression of the house-keeping gene HPRT. Data are shown as mean ± SEM, *n* = 6–9 per experimental group, ^∗^*p* < 0.05 vs. control.

To analyze the regulation of nAchR subunits in patients with DD compared to controls, mRNA expression levels of nAchR subunits α3, α5, α7, β2, and β4 were investigated by qPCR in the tunica muscularis. Expression levels of α3, α5, and β4 fall in the same range (Δ*C*_*t*_ values), whereas subunits β2 and α7 exhibited a 23.5- and 2-fold lower mRNA expression respectively compared to α3, α5, or β4 subunits (data not shown). Whereas nAchR subunit β4 expression was significantly down-regulated in patients with DD, as mRNA levels dropped to 46% ± 7 of control values (Figure 2B), subunits β2, α3, α5, and α7 showed no statistically significant alterations (Figures 2A,C–E).

**FIGURE 3:**
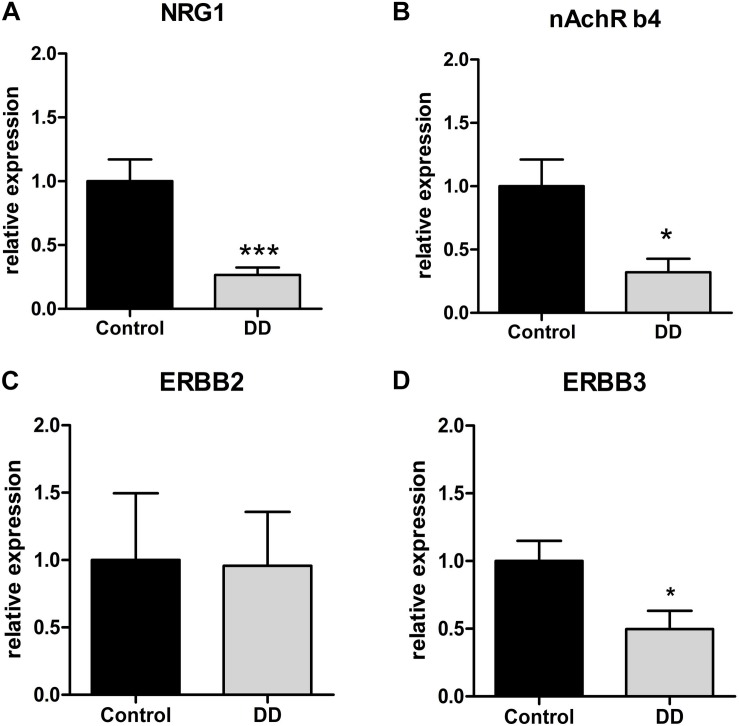
Site-specific mRNA expression of the NRG1 system and nAchRb4 in myenteric ganglia of controls and patients with DD. NRG1 **(A)** and nAchRb4 **(B)** and ErbB3 **(D)** is site-specifically down-regulated in LMD-isolated myenteric ganglia of patients with DD compared to controls. ErbB2 **(C)** exhibited no significant difference. mRNA expression levels are normalized to mRNA expression of the house-keeping gene HPRT. Data are shown as mean ± SEM, *n* = 7–9 per experimental group. ^∗^*p* < 0.05 vs. control, ^∗∗∗^*p* < 0.001 vs. control.

Confirmatory to gene expression analysis in the tunica muscularis, site-specific gene expression in myenteric ganglia retrieved by LMD revealed a significant down-regulation of NRG1, ErbB3 and nAchR β4 in patients with DD by 26 ± 6% (Figure 3A), 49 ± 13% (Figure 3B), and 32 ± 10% (Figure 3D) of control values respectively, whereas the expression of ErbB2 remained unaltered (Figure 3C).

### Immunohistochemistry of NRG1, ErbB2, ErbB3, and nAchR β4 in Patients With DD and Controls

**FIGURE 4:**
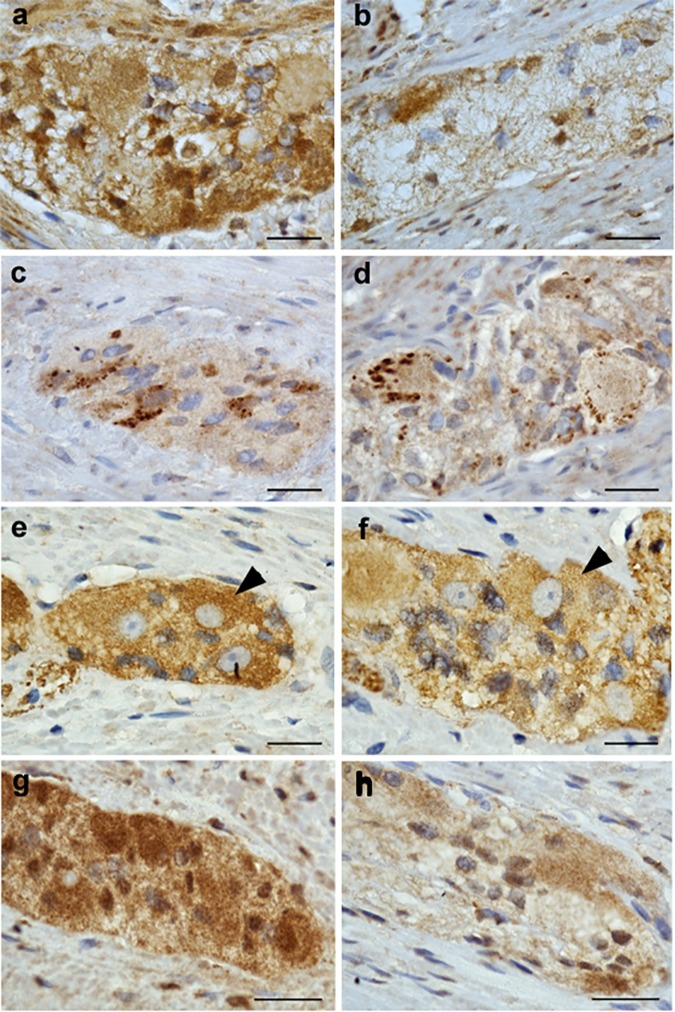
NRG1, ErbB2, ErbB3, and nAchR β4 immunoreactive signals in myenteric ganglia of controls and patients with DD. Robust NRG1 immunoreactive signals (brown colored deposits) detected in myenteric ganglia of controls **(a)** were considerably reduced in a patients with DD **(b)**. The punctuate staining pattern of ErbB2 was equally discernible in both controls **(c)** and patients with DD **(d)**, while ErbB3 immunoreactive signals were decreased in patients with DD **(f)** compared to controls **(e)**, in particular regarding neuronal somata (arrowheads). nAchR β4 immunoreactive signals detected in myenteric ganglia of controls **(g)** are reduced in both neuronal somata and the neuropil of myenteric ganglia from patients with DD **(h)**. Scale bars: 20 μm.

NRG1, ErbB2, ErbB3, and nAchR β4 immunoreactive signals were analyzed in patients with DD and compared to controls. While controls displayed strong NRG1 immunoreactive signals in both neurons and glial cells (Figure 4a), patients with DD showed considerably decreased and patchy immunoreactive signals in myenteric ganglia affecting both neuronal somata and the ganglionic neuropil (Figure 4b). ErbB2 immunoreactivity exhibited a punctuate staining pattern throughout the ganglia with most intensive immunoreactive signals in neuronal somata. No apparent differences between controls (Figure 4c) and patients with DD (Figure 4d) could be observed. In contrast, ErbB3 immunoreactive signals displayed a more homogeneous staining pattern decorating both neuronal somata and the ganglionic neuropil. Compared to controls (Figure 4e), patients with DD (Figure 4f) showed decreased immunoreactive signals for ErbB3, in particular in neuronal somata. nAchR β4 exhibited homogenous immunopositive signals in neuronal somata and the ganglionic neuropil of controls (Figure 4g). In patients with DD, nAchR β4 immunoreactivity was considerably decreased (Figure 4h).

**FIGURE 5:**
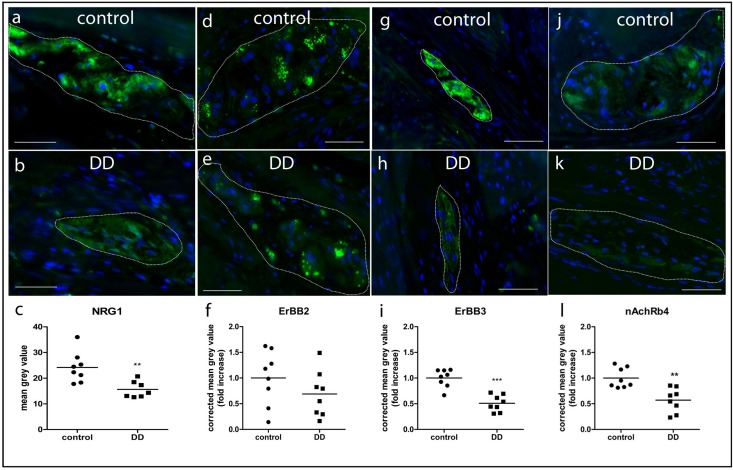
Quantitative analysis of NRG1 **(a–c)**, ErbB2 **(d–f)**, ErbB3 **(g–i)**, and nAchR **(j–l)** β4 immunofluorescent signals in myenteric ganglia of controls and patients with DD. Immunofluorescent signals (green) in myenteric ganglia (marked by white line) of NRG1, ErbB3, and nAchR β4 are significantly reduced in patients with DD compared to controls. Data are shown as corrected mean gray value normalized to controls presented as mean ± SEM, *n* = 8 per experimental group, ^∗∗^*p* < 0.01 vs. control, ^∗∗∗^*p* < 0.001 vs. control. Nuclear stain with DAPI, bars = 50 μm.

The lightmicroscopical findings (Figure 4) were confirmed by subtle quantitative analysis of immunofluorescent signals (Figure 5): patients with DD showed a significant decrease in immunofluorescence intensity of NRG1 to 64 ± 7% (Figures 5a–c), ErbB3 to 50 ± 10% (Figures 5g,h), and nAchRb4 to 57 ± 14% (Figures 5j–l) compared to controls, while immunofluorescence intensity of ErbB2 remained unaltered (Figures 5d–f).

### Effects of NRG1 and GDNF on nAchR α3, α5, α7, β2, β4 mRNA Expression in Cultured Enteric Neurons

**FIGURE 6:**
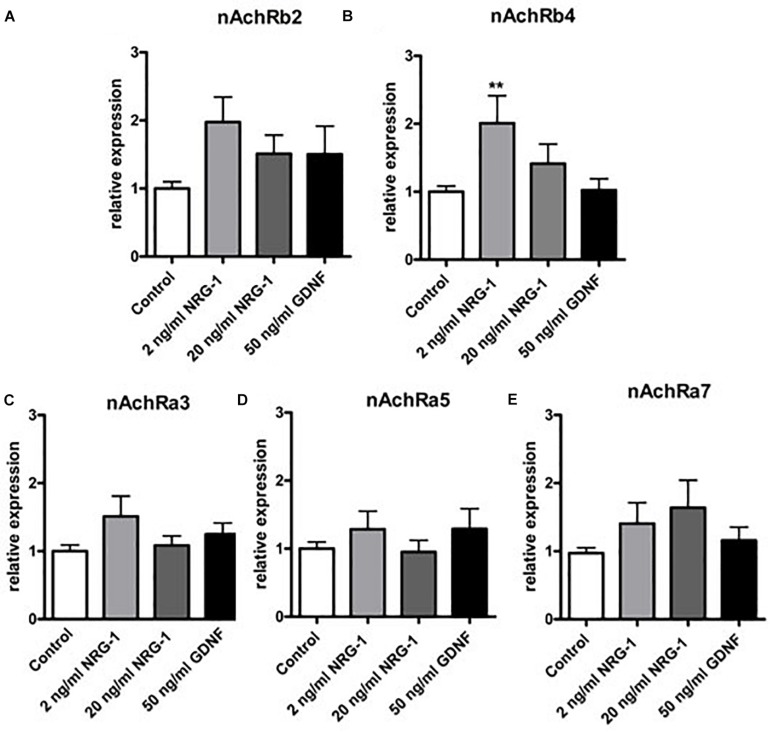
mRNA expression of nAchR receptor subunits in enteric nerve cell cultures after exposure to NRG1 or GDNF. Treatment of cultured enteric nerve cells with NRG1 (2 ng/mL) induced an increased mRNA expression of nAchR subunit β4 **(B)**, while gene expression levels of all other nAchR subunits **(A,C–E)** were not significantly altered. Treatment of GDNF did not show any significant effects on gene expression profiles of nAchR subunits. Expression levels of the target gene were normalized to expression of the house-keeping gene HPRT. Data are shown as mean ± SEM, **n** = 15–18 per experimental group, ^∗∗^**p** < 0.01 vs. control.

To estimate the impact of NRG1 on gene regulation of enteric nAchR subunits, mRNA expression of nAchR subunits α3, α5, α7, β2, and β4 was measured by RT-qPCR in rat enteric nerve cell cultures exposed to NRG1. Effects evoked by NRG1 were compared to those induced by the well characterized enteric neurotrophic factor GDNF. While treatment with NRG1 had no effect on gene expression of the nAchR subunit α3, α5, α7, and β2 (Figures 6A,C–E), NRG1 (2 ng/mL) induced a twofold increase of mRNA expression levels of nAchRβ4 (Figure 6B). Higher concentrations (20 ng/mL) showed not significant increase. GDNF had no effect on gene expression of all nAchR subunits measured (Figures 6A–E).

## Discussion

To our knowledge, this is the first study addressing deficits of the NRG1-system and altered nAchR subunit composition in patients with DD and investigating *in vitro* possible links between NRG1 and nAchR expression in the ENS. The study reveals three important findings: (1) The expression of NRG1 and its corresponding receptor ErbB3 is down-regulated in enteric ganglia and intestinal musculature of patients with DD compared to controls; (2) Whereas the expression of nAchR subunit β4 is decreased in patients with DD, other nAchR subunits remain unaltered; (3) NRG1 treatment promotes the mRNA expression of nAchR subunit b4 in enteric nerve cell cultures.

### Neurotrophic Factors and Their Corresponding Receptors in DD

The pathogenesis of DD is considered to be multifactorial. Increasing evidence is given that DD is associated with an enteric neuropathy (Simpson et al., 2009) characterized by remodeling of nerve tissue (Golder et al., 2003), impaired intestinal motility patterns (Gallego et al., 2013), disturbed enteric neurotransmission (Espin et al., 2014), and a relative loss of enteric nerve cells (Iwase et al., 2005; Deduchovas et al., 2008; Wedel et al., 2010). It has been shown that GDNF, a potent growth factor for the ENS, is impaired not only in DD (Böttner et al., 2013), but also in asymptomatic diverticulosis suggesting that a deficient GDNF system may trigger the enteric nerve cell decrease in DD (Barrenschee et al., 2017).

Neuregulin 1 is also a known growth factor for the ENS. Whereas GDNF is mainly produced by the enteric musculature (Böttner et al., 2013), NRG1 is primarily expressed by enteric neuronal tissue (Barrenschee et al., 2015). Interestingly, GDNF stimulates the expression of NRG1 in motor neurons both *in vitro* and *in vivo* (Loeb and Fischbach, 1997; Loeb et al., 2002), and it is known that locally applied GDNF induces the release of NRG1 from neurons and their axons (Esper and Loeb, 2009). Although in a previous study on enteric nerve cell cultures, we could not confirm that GDNF enhances the gene expression of NRG1 (Barrenschee et al., 2015), it is suggestive that the decreased NRG1 protein expression observed in DD is a consequence of a deficient GDNF system previously described in DD.

Down-regulation of enteric neurotrophic factors and of their corresponding receptors in patients with DD was first investigated by Böttner et al. (2013), who observed decreased gene expression levels of the receptors for GDNF, RET, and GFRα1. A refined analysis revealed that the down-regulation of both receptors was most pronounced in myenteric ganglia (Barrenschee et al., 2017), thereby resembling the actual data on the NRG1-ErbB2/ErbB3 system in DD: The ErbB3 receptor predominantly expressed in enteric neuronal tissue is diminished in DD, whereas the ErbB2 receptor primarily expressed by enteric smooth muscle cells (Barrenschee et al., 2015) is not affected.

It is important to note that only a complex of both receptors can lead to phosphorylation and subsequent signaling in neuronal cells, since ErbB3 lacks kinase domain and ErbB2 lacks a ligand binding site (Holbro et al., 2003). Although not proven in detail by means of phosphorylation studies, the reduction of ErbB3 both at mRNA and protein level suggests a disturbed NRG1-ErbB2/ErbB3 signaling in enteric neuronal tissue. Thus, our findings indicate that the enteric neuropathy associated with DD is characterized by an altered receptor status of several enteric neurotrophic growth factors which in turn may lead to a disturbed signaling in enteric neuronal tissue.

In addition, the data suggest that the loss of GDNF not only leads to the down-regulation of its own receptors (Böttner et al., 2013), but may also cause down-regulation of NRG1 and ErbB3 in enteric neuronal tissue. A functional interaction between NRG1 and GDNF signaling was observed previously by Gui et al. (2013), who found a down-regulation of ErbB3 mRNA expression in response to GDNF stimulation. We could confirm a down-regulation of ErbB3 in enteric nerve cell cultures induced by GDNF and NRG1 (Barrenschee et al., 2015).

It is likely that deficiency of the GDNF system in patients with asymptomatic and symptomatic DD results in a loss of myenteric neurons which in turn leads to a loss of NRG1 expression. The decrease in NRG1 being a potent neurotrophic factor of the ENS and the concomitant down-regulation of its ErbB3 receptor further impairs the maintenance of enteric neuronal tissue and, thus, contributes to the enteric neuropathy in DD. Further studies are required to unravel whether a deficiency of the NRG1/ErbB2/ErbB3 system observed in DD may also occur in asymptomatic diverticulosis.

### Enteric Expression and Regulation of nAchR Subunits in DD

Since nAchRs are critical for gastrointestinal motility (Galligan and North, 2004) and DD is characterized by disturbed intestinal motility (Parks and Connell, 1969; Wedel et al., 2015), we investigated the mRNA expression of those nAchR subunits known to be present in the ENS: the ligand binding nAchR subunits α3, α5, α7 and the structural nAchR subunits β2, β4. While the structural nAchR subunit β4 was down-regulated both at gene and protein level in patients with DD compared to controls, the other subunits remained unaffected.

In autonomic ganglia, the α3β4 subunit combination is considered to be the most prevalent combination (Park et al., 2006). This pattern seems to be conserved as Garza et al. (2009) observed in the ENS of neonatal rats by *in situ* hybridization that the majority of heteromeric nAchRs are also composed of α3β4 (with or without α5), while only a few consist of α3 and β2 subunits. In line with Garza et al. (2009), we also found a lower β2 subunit mRNA expression when compared to that of β4. Noteworthy their semi-quantitative study on the expression of nAchR subunits α3, α5, α7, β2, β4 in the large intestine of the rat matched with our observations.

We found a diminished myenteric β4 subunit expression in patients with DD when compared to control. Since it is known that deficiency of the nAchR subunit β4 results in reduced ileal contractile response to nicotinic agonists in mice (Wang et al., 2003), a dysfunctional nAchR receptor may contribute to the impairment of gastrointestinal motility observed in DD. A compensation of structural subunit β4 to β2 with altered activation kinetics or reduced sensitivity to agonists and antagonist previously described in nerve cell cultures (Nelson et al., 2001; Wang et al., 2003) is rather unlikely, since there is no significant change in the expression of the structural subunit β2 in DD.

In our cell culture experiments, we observed a significant upregulation of nAchR β4 upon NRG1 stimulation, whereas other subunits remained unaffected. This finding is similar to that from Kim et al. (2013), who reported that NRG1 up-regulated the expression of the nAchR α3 and β4 subunits in both sympathetic and parasympathetic major pelvic ganglion neurons from adult rats. In addition, NRG1 was shown to be involved in the formation of nicotinic synapses by increasing the expression of nAchR subunits α3, α5, α7, and β4 in embryonic chick sympathetic neurons (Yang et al., 1998). However, we did not found any other receptor subunits regulated by NRG1, suggesting, that in the human ENS only the β4 subunit is regulated by this growth factor.

## Conclusion

Taken together, our results give first evidence that DD is associated with a decrease of the neurotrophic factor NRG1 and its corresponding receptor ErbB3. The *in vitro* data illustrate that NRG1 promotes the expression of the nAchR β4 subunit, that is also down-regulated in DD suggesting a link between NRG1 and nAchRβ4 expression. Thus, lack of NRG1 in DD may lead to a decrease of nAchRβ4 and subsequently a reduced receptor configuration which in turn may contribute to the impaired intestinal motility observed in DD. Obviously, the enteric neuropathy previously described in DD also involves deficiency of several enteric neurotrophic factors and of neurotransmitter receptors.

## Data Availability Statement

The raw data supporting the conclusions of this article will be made available by the authors, without undue reservation, to any qualified researcher.

## Ethics Statement

The studies involving human participants were reviewed and approved by the Local Ethics Committee of the Faculty of Medicine, Christian-Albrechts University of Kiel, Germany. The patients/participants provided their written informed consent to participate in this study. The animal study was reviewed and approved by the Local Ethics Committee of the Faculty of Medicine, Christian-Albrechts University of Kiel.

## Author Contributions

MBa was responsible for study design, acquisition and interpretation of data, data analysis, and writing of the manuscript. FC was responsible for acquisition and interpretation of data, and critically revising of the manuscript. J-HE and TB contributed to the acquisition of the human material. TW and MBö critically revised the manuscript and wrote the grants financing the study.

## Conflict of Interest

The authors declare that the research was conducted in the absence of any commercial or financial relationships that could be construed as a potential conflict of interest.

## Footnotes

1 antibodies-online.com

2 http://rsbweb.nih.gov/ij/plugins/
